# The chromosomal genome sequence of the giant barrel sponge, *Xestospongia muta* Schmidt 1870 and its associated microbial metagenome sequences

**DOI:** 10.12688/wellcomeopenres.24173.1

**Published:** 2025-07-08

**Authors:** Jose Victor Lopez, Shirley A. Pomponi, Ute Hentschel, Dirk Erpenbeck, Nina Pruzinsky, Cara Fiore, Rebecca Mulheron, Graeme Oatley, Elizabeth Sinclair, Eerik Aunin, Noah Gettle, Camilla Santos, Michael Paulini, Haoyu Niu, Victoria McKenna, Rebecca O’Brien, Wellcome Sanger Institute Tree of Life Management, Samples and Laboratory Team, Wellcome Sanger Institute Scientific Operations: Sequencing Operations, Wellcome Sanger Institute Tree of Life Core Informatics Team, EBI Aquatic Symbiosis Genomics Data Portal Team, Aquatic Symbiosis Genomics Project Leadership

**Affiliations:** 1 National Coral Reef Institute, Department of Biological Sciences, Nova Southeastern University, Dania Beach, Florida, USA; 2 Florida Atlantic University, Boca Raton, Florida, USA; 3 GEOMAR Helmholtz Centre for Ocean Research Kiel, Kiel, Germany; 4 Department of Earth and Environmental Sciences, Ludwig-Maximilian University of Munich, Munich, Germany; 5 Science & Technology Division and Cooperative Institute Support, NOAA Ocean Exploration (UCAR), Silver Spring, Maryland, USA; 6 Appalachian State University, Boone, North Carolina, USA; 7 Tree of Life, Wellcome Sanger Institute, Hinxton, England, UK

**Keywords:** Xestospongia muta, Caribbean barrel sponge, genome sequence, chromosomal, Haplosclerida; microbial metagenome

## Abstract

We present a genome assembly from a specimen of *Xestospongia muta* (Caribbean barrel sponge; Porifera; Demospongiae; Haplosclerida; Petrosiidae). The genome sequence has a total length of 158.52 megabases. Most of the assembly (99.56%) is scaffolded into 15 chromosomal pseudomolecules. The mitochondrial genome has also been assembled and is 18.99 kilobases in length. Several symbiotic bacterial genomes were assembled as MAGs, including *Candidatus* Poribacteria species, Candidatus Latescibacteria, Acidobacteriota, Actinomycetota Gemmatimonadota, multiple Chloroflexota and the archaeon Nitrosopumilus. Gene annotation of this assembly on Ensembl identified 20,220 protein-coding genes.

## Species taxonomy

Eukaryota; Opisthokonta; Metazoa; Porifera; Demospongiae; Heteroscleromorpha; Haplosclerida; Petrosiidae; *Xestospongia*; *Xestospongia muta* (Schmidt, 1870) (NCBI:txid178552)

## Background

**Figure 1.**
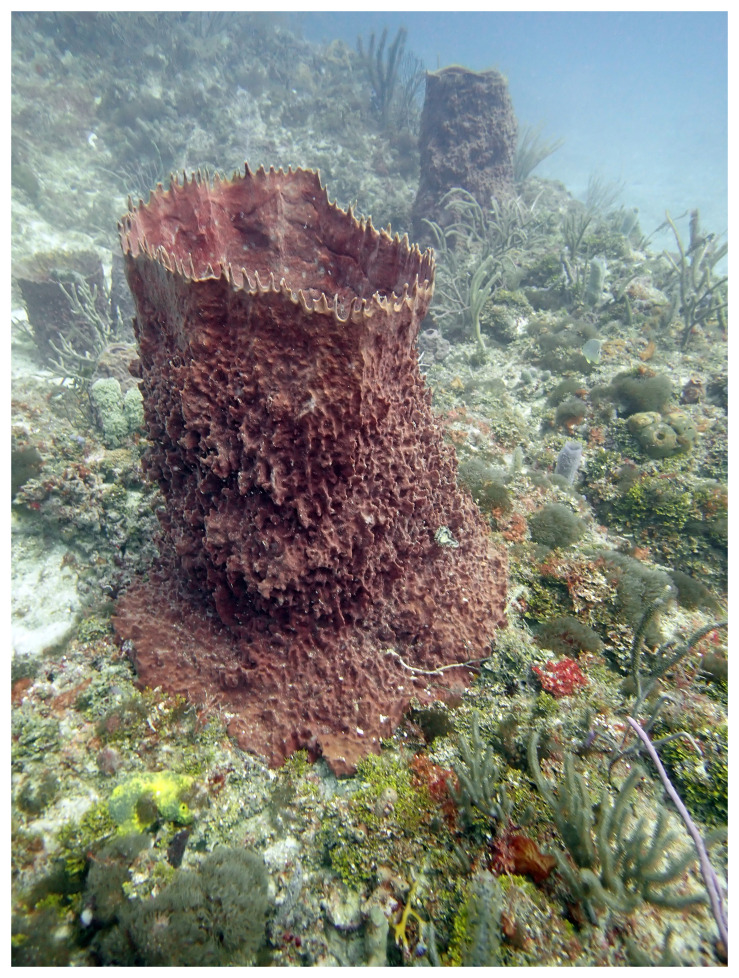
This photograph of a representative *Xestospongia muta* (odXesMuta1 was taken by Brian K Walker on 22 May 2024 at 25 m depth off of Dr. Von D. Mizell-Eula Johnson State Park in Dania Beach, FL.

For a sessile marine invertebrate, the giant barrel sponge species, *Xestospongia muta* Schmidt, 1870, can loom large as one of the most iconic species on Caribbean tropical reefs. These sponges have been nicknamed “redwoods of the reef” because of the massive sizes and their long lifespans, dated to live between hundreds of years to up 2,300 years, comparable to California redwoods ( McMurray *et al.*, 2008). Individual barrel sponges can exceed 2 m in height and 3 m in diameter. The sponge mesohyl is rigid in texture and the outer, light-exposed colour ranges from reddish-brown to purple and grey ( Figure 1). The external surface is irregular with jagged ridges and the spaces between the ridges have a smooth texture. Ostia and oscula (incurrent and excurrent pores) are not visible to the naked eye.

*Xestospongia muta* occurs on many reefs throughout the Caribbean, from the Florida Keys and northern South America to the Lesser Antilles and Trinidad and Tobago. Population densities may reach 0.28 sponge/m ^2^, with demographics on an upward trend in the Florida Keys ( McMurray *et al.*, 2011). With the large biomass and frequent abundance, this sponge species provides key ecosystem services. For example, at reefs where stony coral populations have severely deteriorated, the barrel sponge provides shelter, rugosity, and overall topography. *X. muta* also contributes bioavailable inorganic nitrogen as nitrate to the water column ( Southwell *et al.*, 2008) and may serve as harbingers for reef health, having experienced their own specific marine disease outbreaks ( Angermeier *et al.*, 2011; McMurray *et al.*, 2011). This disease, termed sponge orange band (SOB) disease, on *X. muta* appeared in south Florida in 2012 ( Mulheron, 2014). This event preceded by about two years the more devastating stony coral loss tissue disease (SCTLD) which struck the same geographic region ( Hayes *et al.*, 2022; Lopez, 2023; Rosales *et al.*, 2023).

With the sturdy skeleton of *X. muta* and the presence of spicules as physical defenses, the species may be less reliant on chemical defences. Still, characteristic compounds, including unsaturated polyacetylenic brominated acids ( Patil *et al.*, 1992), the sterols xestosterol and mutasterol ( Li *et al.*, 1981), the chiral tetrahydrofurans, and the mutafurans A–G with antifungal activities were isolated from *X. muta* ( Morinaka *et al.*, 2007). In terms of reproduction, the giant barrel sponge is dioecious, but not sexually dimorphic in the sense that the separate male and female sponges cannot be distinguished from one another. *X. muta* spawns its egg or sperm directly into the water column. This conspicuous event catches divers’ eyes and has been appropriately dubbed “smoking sponges”. Annual, lunar and diel cycles all play a role in the heavily synchronized spawning events ( Neely & Butler, 2020).

*Xestospongia muta* possesses a dense and diverse symbiotic community that is characteristic of high microbial abundance (HMA) sponges ( Fiore *et al.*, 2013; Slaby *et al.*, 2019; Thomas *et al.*, 2016). Fiore *et al*. (2015) carried out one of the first transcriptome studies of *X. muta* that revealed active metabolic expression of multiple nitrogen transformation genes (e.g. archaeal *amo*A) and further identified many active bacterial lineages of the HMA characteristic prokaryotic phyla Gammaproteobacteria, Chloroflexi, Poribacteria, Actinobacteria (in particular, Acidimicrobiales) and Archaea (Nitrosopumilaceae). Cyanobacteria of the *Synechococcus* clade are present inter- and intracellularly in the peripheral sponge tissues, giving the animal its characteristic reddish-brown colouration ( Erwin & Thacker, 2007). Sponge microbiomes may also serve as sensitive indicators for marine environmental stressors such as ocean acidification and climate change ( Lesser *et al.*, 2016; Morrow *et al.*, 2016). Thus, additional genomic information for the microbiomes as well as the host are critical for improving our understanding of the ecology and evolution of sponges. The high-quality chromosomal genome sequence of the Florida *X. muta* and its Australian sister species *X. berqquistia* (GCA_963965975.1) now forms a stable foundation for further studies on this charismatic sponge species’ evolution, ecology and underpinnings of sponge-microbe symbiosis.

## Genome sequence report

### Sequencing data

The genome of a specimen of *Xestospongia muta* ( Figure 1) was sequenced using Pacific Biosciences single-molecule HiFi long reads, generating 83.29 Gb (gigabases) from 6.72 million reads. Based on the estimated genome size, the sequencing data provided approximately 72.0x coverage of the genome. Chromosome conformation Hi-C data produced 103.39 Gb from 684.73 million reads. Table 1 summarises the specimen and sequencing information.

**Table 1.** Specimen and sequencing data for *Xestospongia muta*.

Project information			
**Study title**	Xestospongia muta (giant barrel sponge)		
**Umbrella BioProject**	PRJEB63657		
**Species**	*Xestospongia muta*		
**BioSpecimen**	SAMEA9267624		
**NCBI taxonomy ID**	178552		
Specimen information			
**Technology**	**ToLID**	**BioSample accession**	**Organism part**
**PacBio long read sequencing**	odXesMuta1	SAMEA9267634	somatic animal tissue
**Hi-C sequencing**	odXesMuta1	SAMEA9267633	somatic animal tissue
**RNA sequencing**	odXesMuta3	SAMEA111491447	somatic animal tissue
Sequencing information			
**Platform**	**Run accession**	**Read count**	**Base count (Gb)**
**Hi-C Illumina NovaSeq 6000**	ERR12321227	6.85e+08	103.39
**PacBio Sequel IIe**	ERR11641085	1.85e+06	14.43
**PacBio Sequel IIe**	ERR11641084	4.87e+06	68.86
**RNA Illumina NovaSeq X**	ERR13669939	5.76e+06	0.87

### Assembly statistics

The primary haplotype was assembled, and contigs corresponding to an alternate haplotype were also deposited in INSDC databases. The assembly was improved by manual curation, which corrected 84 misjoins or missing joins and removed 28 haplotypic duplications. These interventions reduced the total assembly length by 4.11% and decreased the scaffold count by 15.3%. The final assembly has a total length of 158.52 Mb in 298 scaffolds, with 186 gaps, and a scaffold N50 of 10.61 Mb ( Table 2).

**Table 2.** Genome assembly data for *Xestospongia muta*.

Genome assembly	
Assembly name	odXesMuta1.1
Assembly accession	GCA_963693285.1
*Alternate haplotype accession*	*GCA_963693275.1*
Assembly level for primary assembly	chromosome
Span (Mb)	158.52
Number of contigs	484
Number of scaffolds	298
Longest scaffold (Mb)	35.36
Assembly metric	Measure
Contig N50 length	1.87 Mb
Scaffold N50 length	10.61 Mb
Consensus quality (QV)	Primary: 56.3; alternate: 49.1; combined 49.7
BUSCO *	C:83.7%[S:82.4%,D:1.4%],F:5.7%,M:10.6%,n:954
Percentage of assembly mapped to chromosomes	99.57%
Organelles	Mitochondrial genome: 18.99 kb

* BUSCO scores based on the metazoa_odb10 BUSCO set using version 5.5.0. C = complete [S = single copy, D = duplicated], F = fragmented, M = missing, n = number of orthologues in comparison.

**Figure 2.**
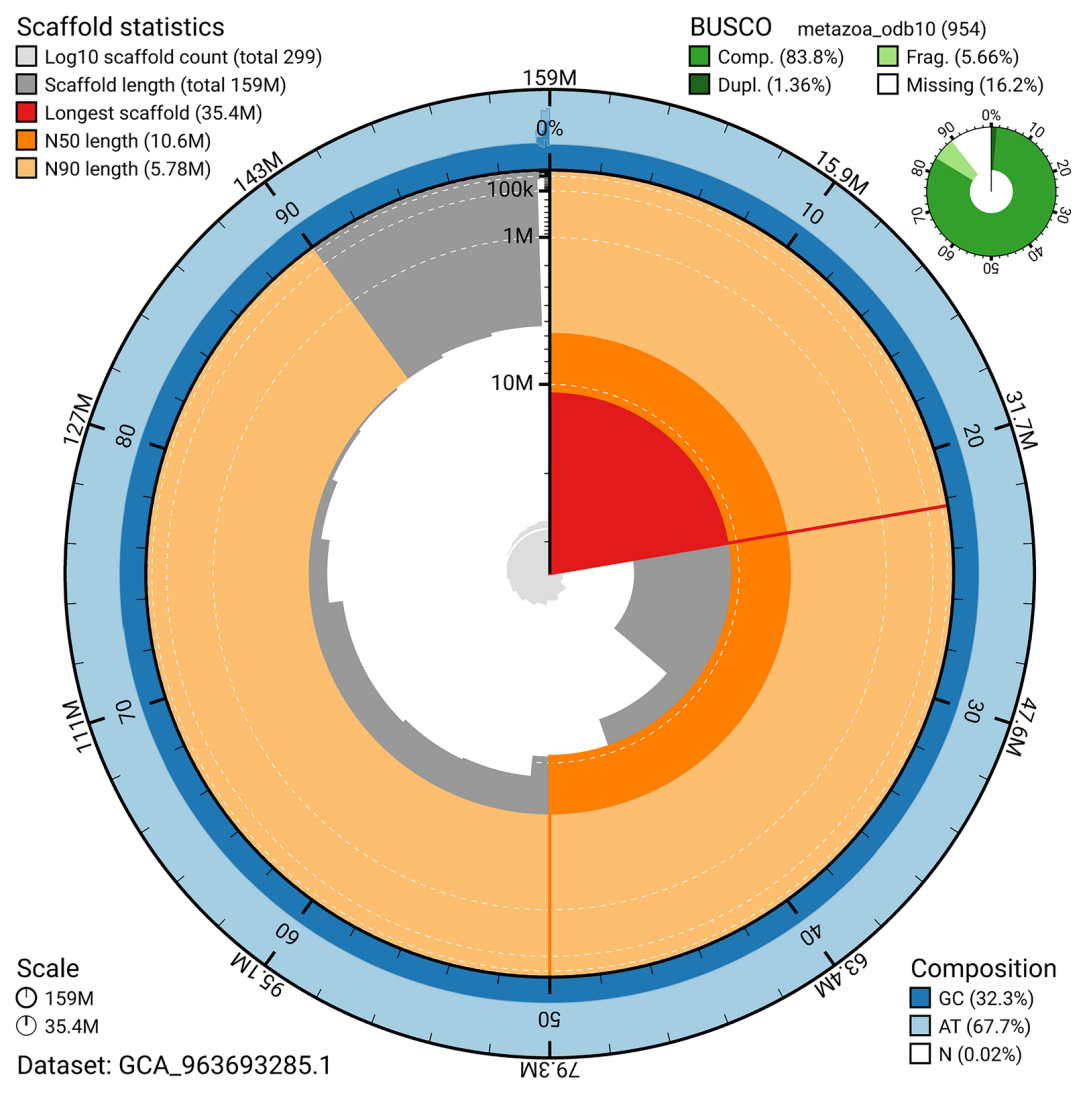
Genome assembly of *Xestospongia muta*, odXesMuta1.1: metrics. The BlobToolKit snail plot provides an overview of assembly metrics and BUSCO gene completeness. The circumference represents the length of the whole genome sequence, and the main plot is divided into 1,000 bins around the circumference. The outermost blue tracks display the distribution of GC, AT, and N percentages across the bins. Scaffolds are arranged clockwise from longest to shortest and are depicted in dark grey. The longest scaffold is indicated by the red arc, and the deeper orange and pale orange arcs represent the N50 and N90 lengths. A light grey spiral at the centre shows the cumulative scaffold count on a logarithmic scale. A summary of complete, fragmented, duplicated, and missing BUSCO genes in the metazoa_odb10 set is presented at the top right. An interactive version of this figure is available at https://blobtoolkit.genomehubs.org/view/GCA_963693285.1/dataset/GCA_963693285.1/snail.

**Figure 3.**
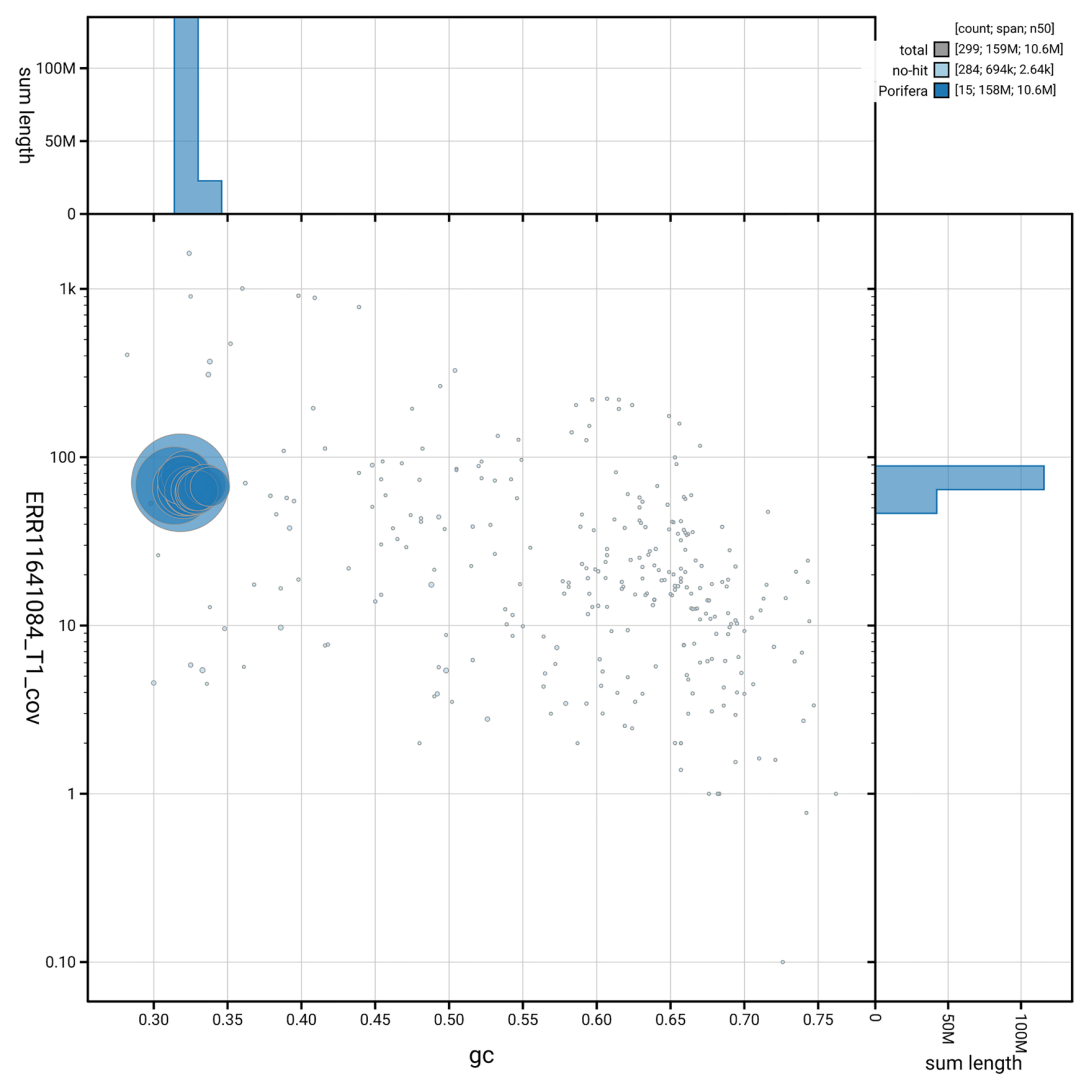
Genome assembly of *Xestospongia muta*, odXesMuta1.1: BlobToolKit GC-coverage plot. Blob plot showing sequence coverage (vertical axis) and GC content (horizontal axis). The circles represent scaffolds, with the size proportional to scaffold length and the colour representing phylum membership. The histograms along the axes display the total length of sequences distributed across different levels of coverage and GC content. An interactive version of this figure is available at https://blobtoolkit.genomehubs.org/view/GCA_963693285.1/dataset/GCA_963693285.1/blob.

**Figure 4.**
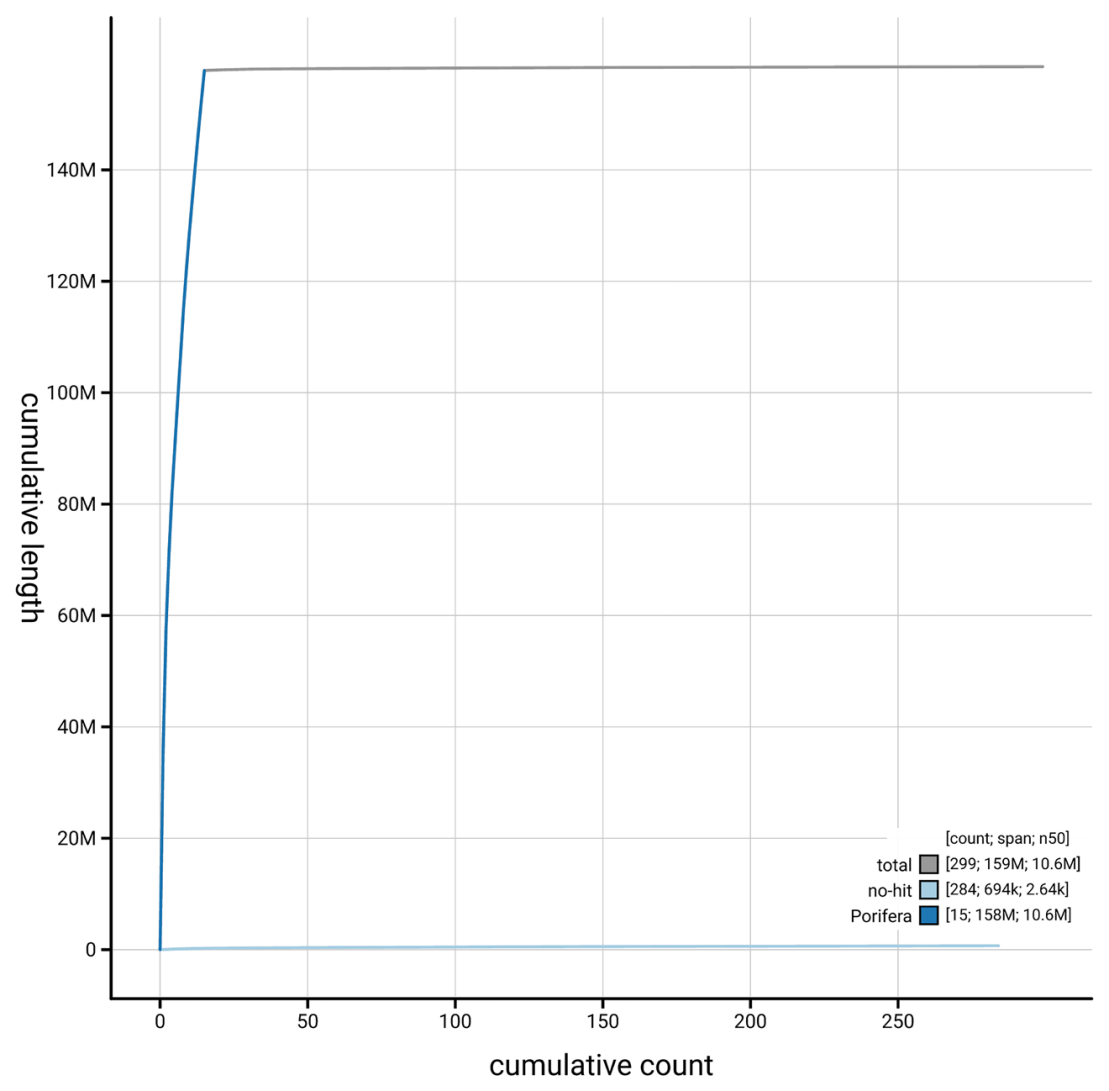
Genome assembly of *Xestospongia muta*, odXesMuta1.1: BlobToolKit cumulative sequence plot. The grey line shows cumulative length for all scaffolds. Coloured lines show cumulative lengths of scaffolds assigned to each phylum using the buscogenes taxrule. An interactive version of this figure is available at https://blobtoolkit.genomehubs.org/view/GCA_963693285.1/dataset/GCA_963693285.1/cumulative.

The snail plot in Figure 2 provides a summary of the assembly statistics, indicating the distribution of scaffold lengths and other assembly metrics. Figure 3 shows the distribution of scaffolds by GC proportion and coverage. Figure 4 presents a cumulative assembly plot, with separate curves representing different scaffold subsets assigned to various phyla, illustrating the completeness of the assembly.

**Figure 5.**
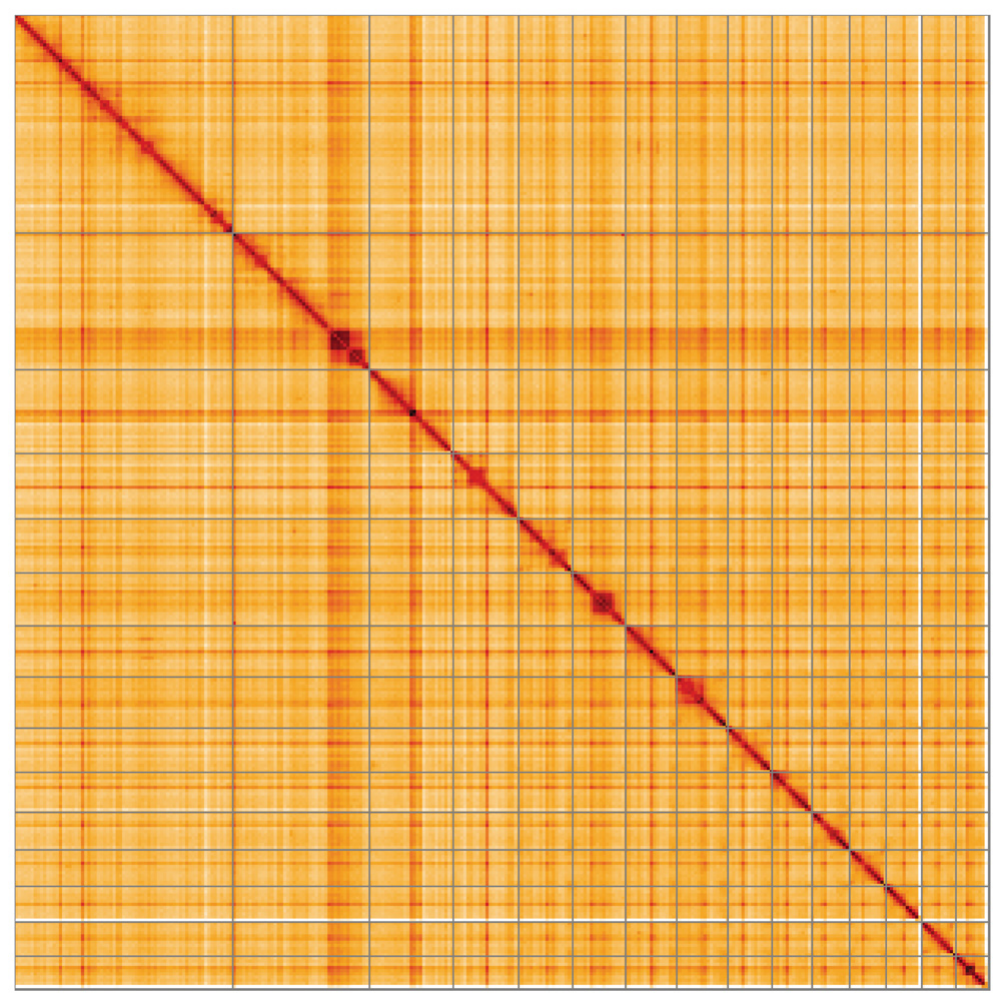
Genome assembly of *Xestospongia muta*: Hi-C contact map of the odXesMuta1.1 assembly, visualised using HiGlass. Chromosomes are shown in order of size from left to right and top to bottom. An interactive version of this figure may be viewed at https://genome-note-higlass.tol.sanger.ac.uk/l/?d=Kkd4NOCzRpiniFavd6eOEg.

Most of the assembly sequence (99.57%) was assigned to 15 chromosomal-level scaffolds. These chromosome-level scaffolds, confirmed by Hi-C data, are named according to size ( Figure 5; Table 3). During curation, it was noted that the exact order of the contigs in the repetitive region of chromosome 2 (16.1–18.9 Mb) is unknown.

**Table 3.** Chromosomal pseudomolecules in the genome assembly of *Xestospongia muta*, odXesMuta1.

INSDC accession	Name	Length (Mb)	GC%
OY848917.1	1	35.36	32
OY848918.1	2	22.15	31.5
OY848919.1	3	13.58	32
OY848920.1	4	10.61	32
OY848921.1	5	8.75	32.5
OY848922.1	6	8.58	32
OY848923.1	7	8.28	32.5
OY848924.1	8	8.28	32
OY848925.1	9	7.16	32.5
OY848926.1	10	6.49	33
OY848927.1	11	6.08	33
OY848928.1	12	5.92	33
OY848929.1	13	5.78	33.5
OY848930.1	14	5.53	33
OY848931.1	15	5.31	34
OY848932.1	MT	0.02	34

The mitochondrial genome was also assembled. This sequence is included as a contig in the multifasta file of the genome submission and as a standalone record in GenBank.

### Assembly quality metrics

The primary haplotype has a QV of 56.3, and the combined primary and alternate assemblies achieve an estimated QV of 49.7. The *k*-mer completeness for the primary haplotype is 58.30%, and for the alternate haplotype it is 85.40%. The combined primary and alternate assemblies achieve a *k*-mer completeness of 90.37%. BUSCO v.5.5.0 analysis using the metazoa_odb10 reference set ( *n* = 954) indicated a completeness score of 83.7% (single = 82.4 %, duplicated = 1.4%).

## Metagenome report

**Figure 6.**
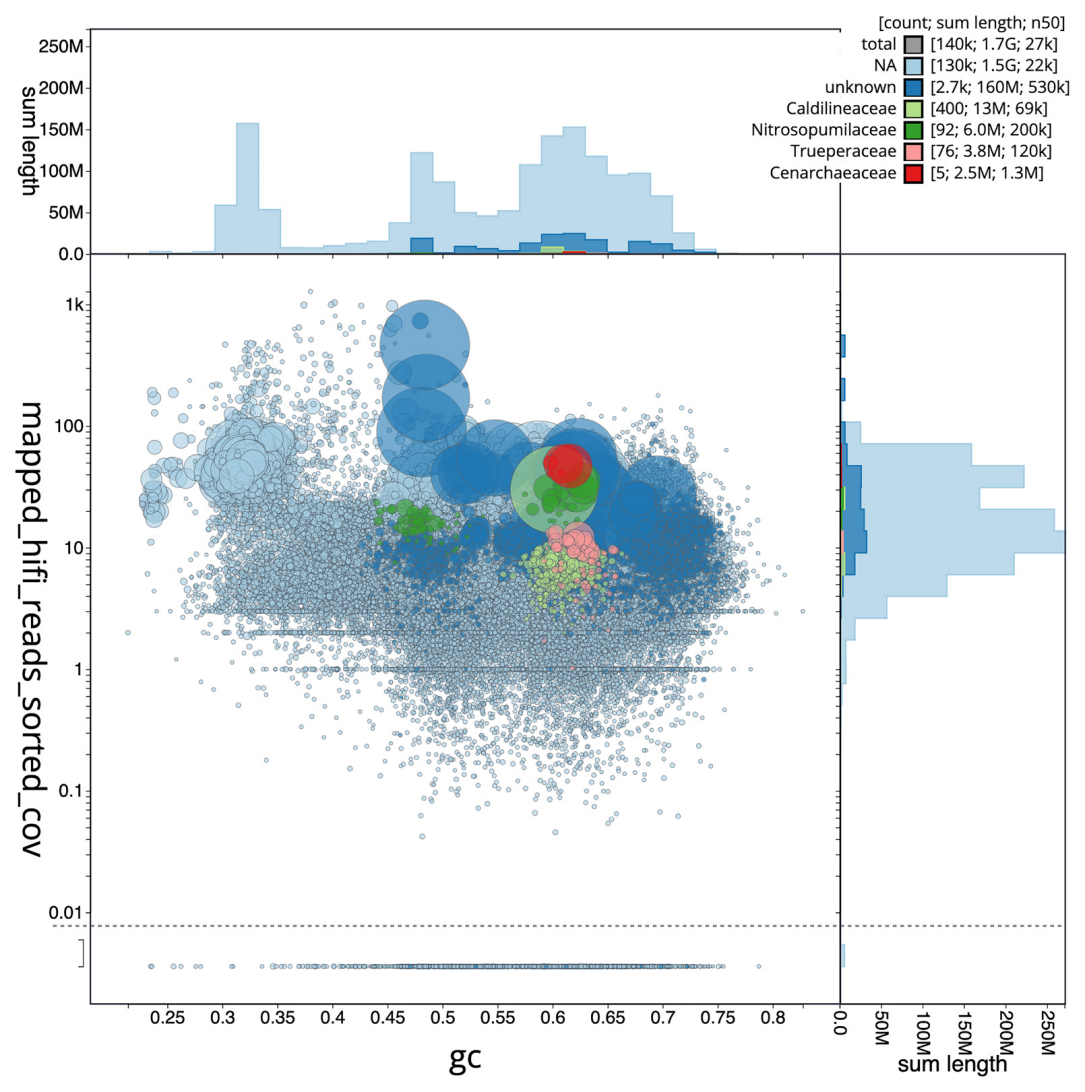
Blob plot of base coverage in mapped against GC proportion for sequences in the *Xestospongia muta* metagenome. Binned contigs are coloured by family. Circles are sized in proportion to sequence length on a square root scale, ranging from 505 to 5,542,969. Histograms show the distribution of sequence length sum along each axis. An interactive version of this figure may be viewed here.

**Figure 7.**
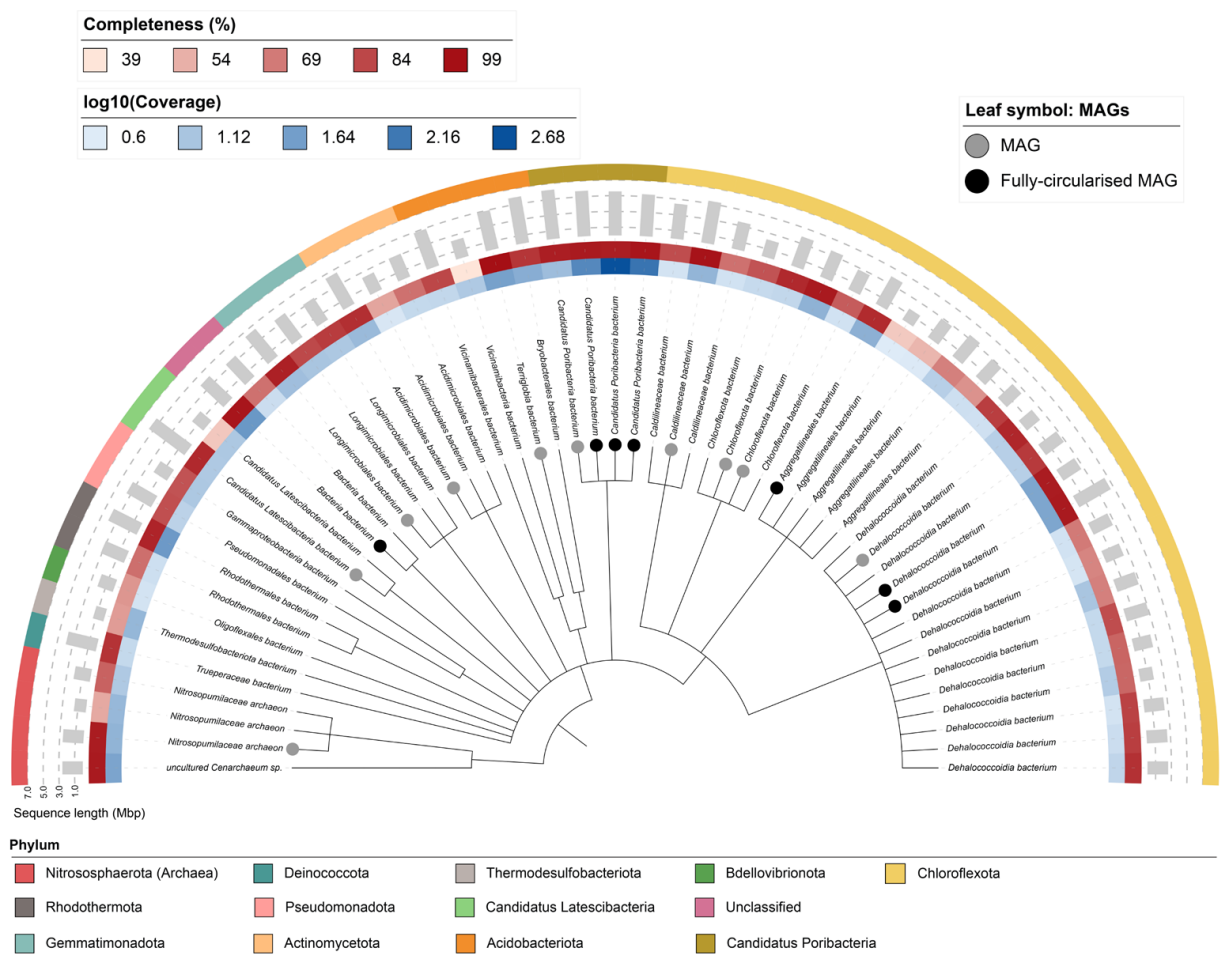
Cladogram showing the taxonomic placement of metagenome bins, constructed using NCBI taxonomic identifiers with *taxonomizr* and annotated in iTOL. Colours indicate phylum-level taxonomy. Additional tracks show sequencing coverage (log _10_), estimated genome size (Mbp), and completeness. Bins that meet the criteria for MAGs are marked with a grey circle; those that are fully circularised MAGs are marked in black.

Fifty-five binned genomes were recovered from the metagenome assembly ( Figure 6), of which 17 were classified as high-quality metagenome-assembled genomes (MAGs) (see Methods). Completeness of the bins ranged from approximately 40% to 99%, with contamination levels below 10%. Detailed information on all binned genomes is available via FigShare ( https://doi.org/10.6084/m9.figshare.28750223). A cladogram is shown in Figure 7.

## Genome annotation report

The *Xestospongia muta* genome assembly (GCA_963693285.1) was annotated at the European Bioinformatics Institute (EBI) on Ensembl Rapid Release. The resulting annotation includes 20,364 transcribed mRNAs from 20,220 protein-coding genes ( Table 2; https://beta.ensembl.org/species/24c58870-89c9-4aa6-84f1-7caeb84c57ec). The average transcript length is 2,765.34, with an average of 6.89 exons per transcript. An alternative annotation is available here:

An alternative annotation for the genome of *Xestospongia muta* performed at the WSI using BRAKER3 produced 24,448 protein-coding genes. This annotation is provided as UCSC assembly hubs and raw downloads at https://github.com/Aquatic-Symbiosis-Genomics-Project/sponge_annotations/tree/main/results/odXesMuta1.

## Methods

### Sample acquisition

Sponge samples were collected by SCUBA at Gulfstream Reef, Boynton Beach FL (latitude 26.51, longitude –80.03) under Florida Fish and Wildlife Conservation Commission fishing licenses and Special Activity License-12-1372-37a. Samples were immediately snap frozen in dry ice baths or stored in TRIZOL preservative before transport to the laboratory at Nova Southeastern University ( Mulheron, 2014). One of the *X. muta* samples for genome sequencing stemmed from the time frame of the SOB outbreak mentioned in the background section, although it was a healthy individual with no signs of disease ( Lopez, 2023). The specimen with ID NSU0023301 (ToLID odXesMuta1) was used for PacBio HiFi sequencing.

The specimen used for RNA sequencing (specimen ID NSU0023303, ToLID odXesMuta3) was collected from the mesophotic reef east of South Carysfort Reef, Key Largo, Florida (latitude 23.25, longitude –80.19) on 2019-08-13 under Florida Keys National Marine Sanctuary Permit FKNMS-2018-175 (to SP). The specimen was collected and identified by Shirley Pomponi (Florida Atlantic University). A fragment of the sponge was dissociated into single cells (containing both sponge cells and symbionts) and cryopreserved using established methods ( Munroe *et al.*, 2018). Cryopreserved cells were thawed rapidly in a 50°C water bath to minimize ice crystal damage to the cells. The cell suspension was rinsed twice by centrifugation at 4000 × rpm for 5 minutes and resuspended in filtered artificial seawater ( Conkling *et al.*, 2019). After the final rinse, the supernatant was decanted and the cell pellet was submitted for sequencing.

### Nucleic acid extraction

The workflow for high molecular weight (HMW) DNA extraction at the Wellcome Sanger Institute (WSI) Tree of Life Core Laboratory includes a sequence of procedures: sample preparation and homogenisation, DNA extraction, fragmentation and purification. Detailed protocols are available on protocols.io ( Denton *et al.*, 2023). The odXesMuta1 sample was prepared for DNA extraction by weighing and dissecting it on dry ice ( Jay *et al.*, 2023). The tissue was cryogenically disrupted using the Covaris cryoPREP ^®^ Automated Dry Pulverizer ( Narváez-Gómez *et al.*, 2023). Prior to DNA extraction, the sponge sample was bathed in “L buffer” (10 mM Tris, pH 7.6, 100 mM EDTA, 20 mM NaCl), minced into small pieces using a scalpel and the cellular interior separated from the mesohyl using forceps ( Lopez, 2022). HMW DNA was extracted using the Automated MagAttract v2 protocol ( Oatley *et al.*, 2023a). For ULI PacBio sequencing, DNA was fragmented using the Covaris g-TUBE method ( Oatley *et al.*, 2023c). Sheared DNA was purified by solid-phase reversible immobilisation, using AMPure PB beads to eliminate shorter fragments and concentrate the DNA ( Oatley *et al.*, 2023b). The concentration of the sheared and purified DNA was assessed using a Nanodrop spectrophotometer and Qubit Fluorometer using the Qubit dsDNA High Sensitivity Assay kit. Fragment size distribution was evaluated by running the sample on the FemtoPulse system.

RNA was extracted from tissue of odXesMuta3 in the Tree of Life Laboratory at the WSI using the RNA Extraction: Automated MagMax™ *mir*Vana protocol ( do Amaral *et al.*, 2023). The RNA concentration was assessed using a Nanodrop spectrophotometer and a Qubit Fluorometer using the Qubit RNA Broad-Range Assay kit. Analysis of the integrity of the RNA was done using the Agilent RNA 6000 Pico Kit and Eukaryotic Total RNA assay.

### Sequencing

Library preparation and sequencing was performed at the WSI Scientific Operations core. Pacific Biosciences HiFi circular consensus DNA sequencing libraries were constructed according to the manufacturers’ instructions for ULI sequencing. Samples were sequenced on a Sequel IIe instrument (Pacific Biosciences, California, USA). Hi-C data were also generated from tissue of odXesMuta1 using the Arima2 kit and sequenced on the Illumina NovaSeq 6000 instrument. Poly(A) RNA-Seq libraries were constructed using the NEB Ultra II RNA Library Prep kit. RNA sequencing was performed on an Illumina NovaSeq X instrument.

### Genome assembly, curation and evaluation

***Assembly***

Prior to assembly of the PacBio HiFi reads, a database of *k*-mer counts ( *k* = 31) was generated from the filtered reads using FastK. GenomeScope2 ( Ranallo-Benavidez *et al.*, 2020) was used to analyse the *k*-mer frequency distributions, providing estimates of genome size, heterozygosity, and repeat content.

The HiFi reads were assembled using Hifiasm ( Cheng *et al.*, 2021) with the --primary option. Haplotypic duplications were identified and removed using purge_dups ( Guan *et al.*, 2020). The Hi-C reads were mapped to the primary contigs using bwa-mem2 ( Vasimuddin *et al.*, 2019). The contigs were further scaffolded using the provided Hi-C data ( Rao *et al.*, 2014) in YaHS ( Zhou *et al.*, 2023) using the --break option for handling potential misassemblies. The scaffolded assemblies were evaluated using Gfastats ( Formenti *et al.*, 2022), BUSCO ( Manni *et al.*, 2021) and MERQURY.FK ( Rhie *et al.*, 2020).

The mitochondrial genome was assembled using MitoHiFi ( Uliano-Silva *et al.*, 2023), which runs MitoFinder ( Allio *et al.*, 2020) and uses these annotations to select the final mitochondrial contig and to ensure the general quality of the sequence.

***Taxonomic verification***

Barrel sponges consist of intertwined species complexes in different ocean basins (see e.g. Swierts *et al*., 2017). *Xestospongia muta* therefore comprise the barrel sponges in the tropical Atlantic, whose monophyletic relationship has not been shown yet. Barrel sponges are currently divided into mitochondrial haplogroups, based on CO1 (I3-M11 fragment) and ATP6. We created separate alignment for both fragments using all published *Xestospongia* spp. sequences of Swierts *et al.* ( Swierts *et al.*, 2017) with using MAFFT v7.450 ( Katoh & Standley, 2013). The current specimen falls into the haplogroup C9A5 sensu, which is currently known from tropical Atlantic barrel sponge specimens only.

***Assembly curation***

The assembly was decontaminated using the Assembly Screen for Cobionts and Contaminants (ASCC) pipeline. Flat files and maps used in curation were generated via the TreeVal pipeline ( Pointon *et al.*, 2023). Manual curation was conducted primarily in PretextView ( Harry, 2022) and HiGlass ( Kerpedjiev *et al.*, 2018), with additional insights provided by JBrowse2 ( Diesh *et al.*, 2023). Scaffolds were visually inspected and corrected as described by Howe *et al*. (2021). Any identified contamination, missed joins, and mis-joins were amended, and duplicate sequences were tagged and removed. The curation process is documented at https://gitlab.com/wtsi-grit/rapid-curation.

***Assembly quality assessment***

The Merqury.FK tool ( Rhie *et al.*, 2020), run in a Singularity container ( Kurtzer *et al.*, 2017), was used to evaluate *k*-mer completeness and assembly quality for the primary and alternate haplotypes using the *k*-mer databases ( *k* = 31) that were computed prior to genome assembly. The analysis outputs included assembly QV scores and completeness statistics.

A Hi-C contact map was produced for the final version of the assembly. The Hi-C reads were aligned using bwa-mem2 ( Vasimuddin *et al.*, 2019) and the alignment files were combined using SAMtools ( Danecek *et al.*, 2021). The Hi-C alignments were converted into a contact map using BEDTools ( Quinlan & Hall, 2010) and the Cooler tool suite ( Abdennur & Mirny, 2020). The contact map is visualised in HiGlass ( Kerpedjiev *et al.*, 2018).

The blobtoolkit pipeline is a Nextflow port of the previous Snakemake Blobtoolkit pipeline ( Challis *et al.*, 2020). It aligns the PacBio reads in SAMtools and minimap2 ( Li, 2018) and generates coverage tracks for regions of fixed size. In parallel, it queries the GoaT database ( Challis *et al.*, 2023) to identify all matching BUSCO lineages to run BUSCO ( Manni *et al.*, 2021). For the three domain-level BUSCO lineages, the pipeline aligns the BUSCO genes to the UniProt Reference Proteomes database ( Bateman *et al.*, 2023) with DIAMOND blastp ( Buchfink *et al.*, 2021). The genome is also divided into chunks according to the density of the BUSCO genes from the closest taxonomic lineage, and each chunk is aligned to the UniProt Reference Proteomes database using DIAMOND blastx. Genome sequences without a hit are chunked using seqtk and aligned to the NT database with blastn ( Altschul *et al.*, 1990). The blobtools suite combines all these outputs into a blobdir for visualisation.

The blobtoolkit pipeline was developed using nf-core tooling ( Ewels *et al.*, 2020) and MultiQC ( Ewels *et al.*, 2016), relying on the Conda package manager, the Bioconda initiative ( Grüning *et al.*, 2018), the Biocontainers infrastructure ( da Veiga Leprevost *et al.*, 2017), as well as the Docker ( Merkel, 2014) and Singularity ( Kurtzer *et al.*, 2017) containerisation solutions.

Table 4 contains a list of relevant software tool versions and sources.

**Table 4.** Software tools: versions and sources.

Software tool	Version	Source
BEDTools	2.30.0	https://github.com/arq5x/bedtools2
bin3C	0.3.3	https://github.com/cerebis/bin3C
BLAST	2.14.0	ftp://ftp.ncbi.nlm.nih.gov/blast/executables/blast+/
BlobToolKit	4.3.3	https://github.com/blobtoolkit/blobtoolkit
BUSCO	5.5.0	https://gitlab.com/ezlab/busco
bwa-mem2	2.2.1	https://github.com/bwa-mem2/bwa-mem2
CheckM	1.2.1	https://github.com/Ecogenomics/CheckM
Cooler	0.8.11	https://github.com/open2c/cooler
DIAMOND	2.1.8	https://github.com/bbuchfink/diamond
dRep	3.4.0	https://github.com/MrOlm/drep
fasta_windows	0.2.4	https://github.com/tolkit/fasta_windows
FastK	427104ea91c78c3b8b8b49f1a7d6bbeaa869ba1c	https://github.com/thegenemyers/FASTK
Gfastats	1.3.6	https://github.com/vgl-hub/gfastats
GoaT CLI	0.2.5	https://github.com/genomehubs/goat-cli
GTDB-TK	2.3.2	https://github.com/Ecogenomics/GTDBTk
Hifiasm	0.16.1	https://github.com/chhylp123/hifiasm
HiGlass	44086069ee7d4d3f6f3f0012569789ec138f42b84aa44357826c0b6753eb28de	https://github.com/higlass/higlass
MAGScoT	1.0.0	https://github.com/ikmb/MAGScoT
MaxBin	2.7	https://sourceforge.net/projects/maxbin/
MerquryFK	d00d98157618f4e8d1a9190026b19b471055b22e	https://github.com/thegenemyers/MERQURY.FK
MetaBat2	2.15-15-gd6ea400	https://bitbucket.org/berkeleylab/metabat/src/master/
MetaTOR	-	https://github.com/koszullab/metaTOR
Minimap2	2.24-r1122	https://github.com/lh3/minimap2
MitoHiFi	2	https://github.com/marcelauliano/MitoHiFi
MultiQC	1.14, 1.17, and 1.18	https://github.com/MultiQC/MultiQC
Nextflow	23.04.1	https://github.com/nextflow-io/nextflow
PretextView	0.2.5	https://github.com/sanger-tol/PretextView
PROKKA	1.14.5	https://github.com/vdejager/prokka
purge_dups	1.2.3	https://github.com/dfguan/purge_dups
samtools	1.18	https://github.com/samtools/samtools
sanger-tol/ascc	-	https://github.com/sanger-tol/ascc
sanger-tol/blobtoolkit	0.3.0	https://github.com/sanger-tol/blobtoolkit
Seqtk	1.3	https://github.com/lh3/seqtk
Singularity	3.9.0	https://github.com/sylabs/singularity
TreeVal	1.2.0	https://github.com/sanger-tol/treeval
YaHS	1.1a.2	https://github.com/c-zhou/yahs

### Metagenome assembly

The metagenome assembly was generated using metaMDBG ( Benoit *et al.*, 2024) and binned using MetaBAT2 ( Kang *et al.*, 2019), MaxBin ( Wu *et al.*, 2014), bin3C ( DeMaere & Darling, 2019), and MetaTOR. The resulting bin sets of each binning algorithm were optimised and refined using MAGScoT ( Rühlemann *et al.*, 2022). PROKKA ( Seemann, 2014) was used to identify tRNAs and rRNAs in each bin, CheckM ( Parks *et al.*, 2015) (checkM_DB release 2015-01-16) was used to assess bin completeness/contamination, and GTDB-TK ( Chaumeil *et al.*, 2022) (GTDB release 214) was used to taxonomically classify bins. Taxonomic replicate bins were identified using dRep ( Olm *et al.*, 2017), with default settings (95% ANI threshold). The final bin set was filtered for bacteria and archaea. All bins were assessed for quality and categorised as metagenome-assembled genomes (MAGs) if they met the following criteria: contamination ≤ 5%, presence of 5S, 16S, and 23S rRNA genes, at least 18 unique tRNAs, and either ≥ 90% completeness or ≥ 50% completeness with fully circularised chromosomes. Bins that did not meet these thresholds, or were identified as taxonomic replicates of MAGs, were retained as ‘binned metagenomes’ provided they had ≥ 50% completeness and ≤ 10% contamination. A cladogram based on NCBI taxonomic assignments was generated using the ‘taxonomizr’ package in R. The tree was visualised and annotated using iTOL ( Letunic & Bork, 2024). Software tool versions and sources are given in Table 4.

### Host genome annotation

The BRAKER2 pipeline ( Brůna *et al.*, 2021) was used in the default protein mode to generate annotation for the *Xestospongia muta* assembly (GCA_963693285.1) in Ensembl Rapid Release at the EBI.

### Wellcome Sanger Institute – Legal and Governance

The materials that have contributed to this genome note have been supplied by a Tree of Life collaborator. The Wellcome Sanger Institute employs a process whereby due diligence is carried out proportionate to the nature of the materials themselves, and the circumstances under which they have been/are to be collected and provided for use. The purpose of this is to address and mitigate any potential legal and/or ethical implications of receipt and use of the materials as part of the research project, and to ensure that in doing so we align with best practice wherever possible. The overarching areas of consideration are:

• Ethical review of provenance and sourcing of the material

• Legality of collection, transfer and use (national and international)

Each transfer of samples is undertaken according to a Research Collaboration Agreement or Material Transfer Agreement entered into by the Tree of Life collaborator, Genome Research Limited (operating as the Wellcome Sanger Institute) and in some circumstances other Tree of Life collaborators.

## Data Availability

European Nucleotide Archive: *Xestospongia muta* (giant barrel sponge). Accession number PRJEB63657; https://identifiers.org/ena.embl/PRJEB63657. The genome sequence is released openly for reuse. The *Xestospongia muta* genome sequencing initiative is part of the Aquatics Symbiosis Genomics (ASG) project. All raw sequence data and the assembly have been deposited in INSDC databases. The genome will be annotated using available RNA-Seq data and presented through the Ensembl pipeline at the European Bioinformatics Institute. Raw data and assembly accession identifiers are reported in Table 1 and Table 2.

## Author information

Members of the Wellcome Sanger Institute Tree of Life Management, Samples and Laboratory Team are listed here: https://doi.org/10.5281/zenodo.10066175.

Members of the Wellcome Sanger Institute Scientific Operations: Sequencing Operations are listed here: https://doi.org/10.5281/zenodo.10043364.

Members of the Wellcome Sanger Institute Tree of Life Core Informatics team are listed here: https://doi.org/10.5281/zenodo.10066637.

Members of the European Bioinformatics Institute ASG Data Portal team are listed here: https://doi.org//10.5281/zenodo.10076466.

Members of the Wellcome Sanger Institute/Aquatic Symbiosis Genomics Project Leadership are listed here: https://doi.org/10.5281/zenodo.10184833.
