# Supplementary material for: The chromosomal genome sequence of the giant barrel sponge, Xestospongia muta Schmidt 1870 and its associated microbial metagenome sequences
Source: Wellcome Open Res. 2025 Jul 8;10:336. [Version 1] doi: 10.12688/wellcomeopenres.24173.1 (PMC12398682; doi:10.12688/wellcomeopenres.24173.1)
Supplement: Peer review history

# Reviewer report: Reviewer response for version 1

**DOI:** 10.21956/wellcomeopenres.26664.r127266

**Published:** 2025-08-30

**Authors:** Arzu Karahan

**Affiliations:** 1 Middle East Technical University, Erdemli-Mersin, Turkey

The research titled 'The chromosomal genome sequence of the giant barrel sponge, Xestospongia muta Schmidt 1870 and its associated microbial metagenome sequences' is a well-designed, prepared, and concluded study. It can be accepted for indexed with minor revision. 

  I only recommend rotating the text on the Y-axis of the graphs upwards.

Are sufficient details of methods and materials provided to allow replication by others?

Yes

Is the rationale for creating the dataset(s) clearly described?

Yes

Are the datasets clearly presented in a useable and accessible format?

Yes

Are the protocols appropriate and is the work technically sound?

Yes

Reviewer Expertise:

metabarcoding, metagenomics, transcriptomics on Tunicates

I confirm that I have read this submission and believe that I have an appropriate level of expertise to confirm that it is of an acceptable scientific standard.

# Reviewer report: Reviewer response for version 1

**DOI:** 10.21956/wellcomeopenres.26664.r128020

**Published:** 2025-08-28

**Authors:** Maximiliano M. Maronna

**Affiliations:** 1 Federal University of Rio Grande, Rio Grande, Brazil

The DATA NOTE submitted by Lopez et al. describes the genome assembly of one individual of *Xestospongia muta*. Main text presents proper details about general biology, biogeography and recent ecological events.

 The applied methodology is a high-quality approach, including long reads, short reads for Hi-C spatial proximity method and  transcriptomic sequencing. This manuscript is well-written and provides information related to materials and methods plus genome assembly, including exogenous DNA removal and metagenome assemblies.

 Minor suggestions to improve this article:

 (1) To detail number of current species for *Xestospongia* in the Background section.

 (2) To better describe the “holobiont transcriptomic profile” in Background section, “carried out one of the first transcriptome studies of *X. muta *that revealed active metabolic expression of multiple nitrogen transformation genes (e.g. archaeal *amo*A)…” The way it is presented in this first version, it would be interpreted that *X. muta *is expressing archaeal genes, among others.

 (3) To describe basic genome heterozygosity, a histogram of k-mer multiplicity from Illumina reads would be easy to calculate and informative as part of main figures.

 Also, I would like to remark two important points from another review I did a few months ago, in this same journal:

 “(5) To create as Supplementary Material a genome report sheet as recommended in Santander et al 2022 ( refer to 1 ). Doing so, it will be easier to learn about all inputs_outputs for each step, and to combine+compare with similar datasets for comparative analyses. Also, an additional document briefly describing workflow steps, scripts and command lines via GitHub or similar (e.g., GitLab) would be very useful. Current format at Github is quite fragmented and not very easy to understand.

 (4) Busco values are not represented in the GenBank datasets section. I understand this is a Data Note, but the relatively medium-low BUSCO values for a chromosome-level assembly would be interesting to remark (maybe this is not an artifact, and this early-branching lineage present a reduced BUSCO elements).”

Are sufficient details of methods and materials provided to allow replication by others?

Yes

Is the rationale for creating the dataset(s) clearly described?

Yes

Are the datasets clearly presented in a useable and accessible format?

Yes

Are the protocols appropriate and is the work technically sound?

Yes

Reviewer Expertise:

Evolutionary Genetics, Marine Genetics, Genomics

I confirm that I have read this submission and believe that I have an appropriate level of expertise to confirm that it is of an acceptable scientific standard.

## References

[1] : The state of Medusozoa genomics: current evidence and future challenges. *GigaScience* .2022;11: 10.1093/gigascience/giac036 10.1093/gigascience/giac036 PMC911276535579552
